# The Impact of Sustainability Courses: Are They Effective in Improving Diet Quality and Anthropometric Indices?

**DOI:** 10.3390/nu16111700

**Published:** 2024-05-30

**Authors:** Çağla Pınarlı Falakacılar, Sevinç Yücecan

**Affiliations:** 1 Department of Nutrition and Dietetics, Institute of Health Sciences, Lokman Hekim University, 06510 Çankaya, Ankara, Turkey; 2 Department of Nutrition and Dietetics, Faculty of Health Sciences, Lokman Hekim University, 06510 Çankaya, Ankara, Turkey; sevinc.yucecan@lokmanhekim.edu.tr

**Keywords:** sustainability, sustainable nutrition, sustainable nutrition education, diet quality, Healthy Eating Index, Mediterranean diet score, anthropometric measurements, university students, carbon footprint, water footprint

## Abstract

There are studies on the effect of general nutrition education on diet quality and anthropometric measurements, while studies showing the effectiveness of sustainable nutrition education, which also addresses the effect of food on the environment, are quite limited. This study aimed to investigate the effects of sustainable nutrition education on diet quality, anthropometric measurements, and the carbon footprint (CFP) and water footprint (WFP) of diet. A total of 160 university students received 1 h of sustainable nutrition education for 6 weeks. Before, at the end of, and 2 months after the courses, 24 h food consumption records were taken to assess diet quality and CFP and WFP values of diet, and Mediterranean diet (MedDiet) and Healthy Eating Index (HEI)-2020 scores were evaluated. The results of the study showed that sustainable nutrition education increased MedDiet score by 1.86 points and HEI-2020 score by 7.38 points. This education program also decreased body weight, body mass index (BMI), fat mass, and neck circumference. Sustainability education has a positive impact on calcium, potassium, and magnesium intakes, a negative impact on vitamin B12 and zinc intakes, and no effect on total protein intake. Education resulted in a 22% reduction in CFP and a 10% reduction in WFP.

## 1. Introduction

The impact of diet on human health and environmental health has gained attention in recent years [1]. Unhealthy dietary patterns, such as the Western-type diet, increase the risk of obesity and related non-communicable diseases. The World Obesity Atlas estimates that 50% of the population will be overweight or obese by 2030 [2]. On the other hand, unhealthy dietary patterns are also associated with increased use of clean water resources and the generation of greenhouse gases, a major cause of global warming. High greenhouse gas emissions also appear to reduce the levels of zinc, iron, B vitamins, and protein in food [3,4]. The European Commission’s report titled “A Clean Planet for All” emphasizes that it is important to change consumers’ food choices to improve health and make greenhouse gas emissions neutral by 2050 [5].

Globally, 2.4 billion people lack access to sufficient food, and approximately 735 million people are hungry [6]. These problems are expected to worsen as the world’s population is expected to reach 8.6 billion by 2030 and 9.8 billion by 2050, posing a significant challenge to providing a sufficient and balanced diet to this population [7].

The concept of sustainable nutrition has come to the forefront as providing a diet with sufficient nutrients for people while at the same time maintaining planetary well-being and is set to become a global challenge [8]. Sustainable nutrition aims not only to promote optimal health but also to minimize environmental impact by aiming to adopt a culturally appropriate, readily available, and environmentally friendly diet. Research shows that there is a significant lack of knowledge about the concept of sustainable nutrition [9,10]. Having sustainable eating behaviors seems to have potential outcomes for both controlling body weight and body fat and reducing the carbon footprint (CFP) and water footprint (WFP) [11,12].

Sustainable dietary patterns are also seen to have high diet quality [13]. Importantly, the relationship between diet quality and environmental sustainability appears to vary depending on how diet quality is measured [14]. Different results can be obtained from different diet quality assessment methods [15,16,17]. Therefore, the use of different diet quality measurement methods is important in assessing the relationship between sustainability and diet quality. Considering the literature, the 14-point Mediterranean Diet Adherence Screener (MEDAS) and the Healthy Eating Index (HEI) stand out as diet quality indicators.

The Mediterranean diet (MedDiet) includes high consumption of olive oil, olives, fruits, vegetables, cereals (especially whole grains), legumes, and oilseeds; moderate to high consumption of fish; moderate consumption of eggs, poultry; and dairy products; and low consumption of red meat and its products and desserts. Research indicates that following the MedDiet can decrease the likelihood of cardiovascular disease and cancer while enhancing cognitive health [18]. The Mediterranean diet was recognized as an “intangible cultural heritage of humanity” by the United Nations Educational, Scientific and Cultural Organization (UNESCO) in 2010. From a sustainability standpoint, the Mediterranean diet offers four notable advantages, including significant health and nutritional benefits, reduced environmental impact and promotion of biodiversity, high sociocultural food value, and favorable local economic outcomes [19]. Adhering closely to the Mediterranean diet appears to lead to weight reduction and a decrease in the prevalence of overweight and/or obesity [20].

Considering the adherence to local dietary guidelines, the HEI is one of the most frequently used methods to assess diet quality [21]. Nutrition education is thought to have potential positive effects on both increasing the HEI score and reducing body weight [22,23].

The years of university education, which are considered young adulthood, constitute an important stage in which dietary habits that may influence nutrition status and overall health in later stages of life are established. Assessment of nutritional quality among university students is of great importance for promoting a healthier lifestyle in the years to come and protecting the environment [24].

It is considered important to follow sustainable dietary behaviors to reduce global warming by using fewer freshwater resources and to improve diet quality and combat obesity. It is seen that general nutrition education has positive effects in changing both diet quality and anthropometric measurements. Apart from general nutrition education, studies evaluating the effects of sustainable nutrition education are quite limited. Students in the 20–25 age group have a lower planetary health diet index, even if they receive general nutrition education [24]. In order to achieve the Sustainable Development Goals and improve both overall public health and planetary health, it is necessary to develop sustainable dietary behaviors. Sustainable nutrition education plays an important role in shaping these patterns [25]. In studies of different groups, it has been shown that in general people believe that diet affects their health but has little or no impact on the environment [26,27]. It has been observed that the main barrier to access to sustainable and healthy food is a lack of awareness [28]. At this point, it is considered necessary to add sustainability education. Planning sustainable nutrition education and evaluating the effect of this education on diet quality, anthropometric measurements and environmental impact will contribute to the literature. 

For all these reasons, the aim of this study is to reveal the effects of sustainable nutrition education on diet quality, anthropometric measurements, and diet-related CFP and WFP.

## 2. Materials and Methods

### 2.1. Study Design and Participants

The cross-sectional intervention study was conducted on students studying at the Faculty of Health Sciences of a foundation university in Istanbul who agreed to participate in the study. There are 4 departments in the Faculty of Health Sciences: Nutrition and Dietetics, Child Development, Physiotherapy and Rehabilitation, and Nursing. 

According to the analysis with 80% power and 0.05% error margin, the minimum sample size was 158 people. To eliminate the difference in vocational education, equal numbers of students were taken from each department and class. The sample of the research consists of 40 students from each department, totaling 160 students. Within the scope of the research, face-to-face sustainable nutrition courses were provided for 1 h per week for 6 weeks between October and November 2023. General nutrition courses that attempt to increase MedDiet adherence or HEI scores typically focus on the health effects of foods, recommendations for food group consumption, and the importance of macronutrients and micronutrients. However, this course aims to measure the effectiveness of an educational approach that, in addition to covering the health effects of foods, also addresses topics such as food waste, food loss, and the environmental impact of food. This approach is designed to go beyond the standard nutrition education by integrating these critical issues.

**Figure 1:**
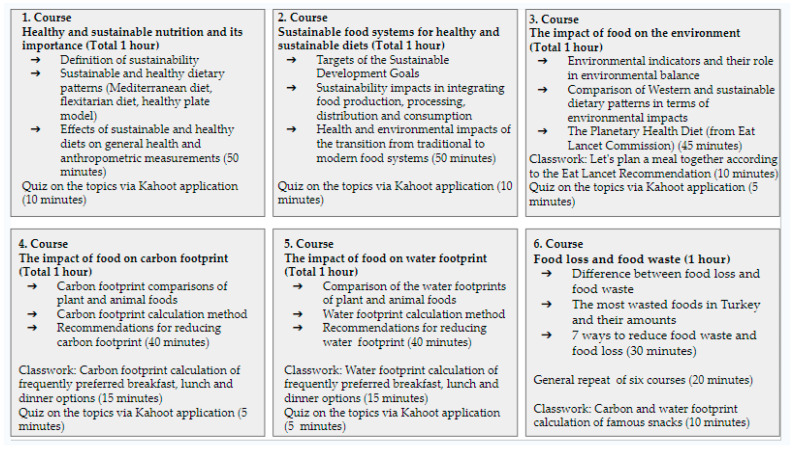
Course Flowchart.

In order to use active learning techniques during the courses, applications such as in-class studies and quiz competitions were carried out. The topics covered in the weeks and practices and their durations (approximately) are given in Figure 1 as “Course Flowchart”.

Data collection tools were administered 3 times in total: before the course, at the completion of the course, and 2 months after the completion of the course.

This study was conducted in accordance with the principles of the Declaration of Helsinki. Ethics committee permission was obtained from Lokman Hekim University Non-Interventional Clinical Research Ethics Committee (Decision No: 2023/6-Code No: 2023006). Students who agreed to participate in the study were educated and the relevant questionnaires were answered. Each participant read and signed an informed consent form at the beginning of study.

### 2.2. Data Collection Methods

At the beginning of the course, students’ information such as gender, age, department, and class were queried. In addition, questions were asked to assess individuals’ awareness of sustainable nutrition.

In terms of anthropometry, bioelectrical impedance analysis was applied using a digital scale (Tanita BC-418 Segmental Body Analyzer, Tanita Corp., Tokyo, Japan) to obtain weight, body fat percentage (%), and fat mass and muscle mass (kg). A fasting period of at least 3 h was required for body weight measurement and body composition analysis, and individuals who were not suitable for bioelectrical impedance analysis were not included in the study [29].

Participants’ height was measured only before the training. The height of the participants was measured with their feet together and against the wall, with their heads in the Frankfort plane, with the eye triangle and the top of the auricle in the same alignment, with a non-flexible tape measure, and without shoes [30].

Neck circumference measurement was used as an indicator of upper body fat distribution. Neck circumference was preferred because it is technically easier to measure than waist circumference. Neck circumference is measured as the circumference passing just below the laryngeal prominence and at the junction of the neck and shoulders. A neck circumference of ≥34 cm in women and ≥37 cm in men is considered a risk factor for chronic diseases, especially obesity [31]. Finally, the body mass index (BMI) of the participants was calculated.

Different methods were used to assess diet quality in the study. One of these methods, the MEDAS questionnaire, consists of a total of 14 questions. The Turkish validity and reliability of the scale was performed by Bekar et al. in 2023. A total score below 7 is known as low adherence to the MedDiet. A total score between 7 to 9 indicates that the individual has moderate adherence to the MedDiet. A total score of 9 and above indicates high adherence to the MedDiet [32].

The HEI-2020, another method used to assess diet quality, was developed in 1995 to assess the extent to which Americans comply with dietary recommendations. This index consists of a total of 13 components, 9 of which are adequacy components and 4 of which should be limited. Adequacy is defined for total fruit, whole fruit, total vegetables, dark green leafy vegetables and legumes, plant-derived proteins and seafood, total protein, milk and dairy products, whole grains, and moderate intake for fatty acids, processed grains, sodium, added sugar, and saturated fat [21]. The HEI-2020 is the same as the HEI-2015 and has been referred to as the HEI-2020 to indicate that the index is still up to date [33]. A 24 h dietary record was used to calculate the HEI score.

The 24 h dietary record was used to determine the students’ energy, macronutrient and micronutrient intakes that changed with the education program. Twenty-four-hour dietary records were obtained by retrospective recall method. In determining the amounts and portions of the foods consumed, the “Food and Nutrition Photo Catalog: Measurements and Quantities” was used [34]. Nutrition Information System (BEBIS) version 9 was used analyze the energy, macronutrient, and micronutrient intakes of students [35]. 

The CFP and WFP were also calculated using a 24 h dietary record. The BEBIS program calculates the calories of foods and distributes them by food group. An Excel worksheet was created to calculate the CFP and WFP values of all foods/food groups obtained from BEBIS. The CFP and WFP dataset specified in Excel version 2404 (Build 17531.20152) was created using the databases listed below.

A database containing the total WFP of plant and animal foods produced in Turkey was primarily used to assess the WFP of the diet [36]. Data from a study conducted in the Mediterranean region, including Turkey, were used for foods for which Turkey-specific data were not calculated [37]. Various studies and data were used to calculate CFP [38,39,40,41,42]. For all other calculations that could not be obtained from these sources, a database containing the WFP and CFP of more than 57,000 food products was used [43]. 

CFP and WFP values have been adjusted to 2000 kcal/day using a special multiplier to facilitate comparison with the literature. The Excel file included in the Supplementary Materials contains the data set and an example of the calculation of CFP and WFP.

All the mentioned assessments were repeated before, at the completion of, and 2 months after the completion of the course.

### 2.3. Statistical Analysis

The data obtained in the study were analyzed using SPSS (Statistical Package for Social Sciences) for Windows 25.0 program. Number, percentage, mean, and standard deviation were used as descriptive statistical methods in the evaluation of the data. The one-way ANOVA test was used to compare quantitative continuous data between more than two independent groups. The Scheffe test was used as a complementary post hoc analysis to determine the differences after the ANOVA. The repeated measures ANOVA test was applied to determine the difference between repeated measures, and the complementary Bonferroni test was applied. In addition, effect sizes for repeated measures were calculated. Eta squared (η^2^) coefficients were used to calculate the effect size. The effect size shows whether the difference between the groups is a large enough difference to be considered significant. The Eta squared value is considered as follows: 0.01: small effect; 0.06: medium effect; 0.14: large effect [44]. The relationships between continuous variables were tested with Pearson correlation analysis. A *p*-value of <0.05 was considered statistically significant.

## 3. Results

### 3.1. Demographic Characteristics of University Students

In this study, which was conducted on a total of 160 students, an equal number of 40 participants (25%) were recruited from each department (Nutrition and Dietetics, Child Development, Nursing, Physiotherapy and Rehabilitation). The mean age of the participants was 21.13 ± 2.31 years, 137 (85.6%) were female, and 23 (14.4%) were male. There were no male students in the Departments of Nutrition and Dietetics and Child Development, 12 male students in the Department of Nursing, and 11 male students in the Department of Physiotherapy and Rehabilitation. Details of the socio-demographic characteristics and sustainability awareness levels of the students are shown in Table 1.

### 3.2. Anthropometric Indices of University Students

Anthropometric evaluations such as body weight, height, BMI, fat percentage, fat mass, muscle mass, and neck circumference were performed. The mean height of the students was 165.48 ± 6.57 cm (Min = 153; Max = 187). Body weight, BMI, fat percentage, and neck circumference decreased significantly compared to pre-course levels, both at the completion of the courses and 2 months afterward (*p* < 0.05) (Table 2).

Fat and muscle mass of the students did not show a significant difference during the education program (*p* > 0.05) (Table 2).

When the anthropometric measurements of the students according to the department of study were analyzed, muscle mass and neck circumference measurements showed a significant difference during the education program (*p* < 0.05). There was no significant difference between body weight, BMI, body fat percentage, and fat mass by department during the education program (*p* > 0.05) (Supplementary Tables S1–S3).

### 3.3. Diet Quality Markers of University Students

#### 3.3.1. MedDiet Scores of Students

The total MedDiet score increased significantly at the end of the course and 2 months after the course compared to the pre-course (*p* < 0.05). The increase between the completion of the course and 2 months after the course was found to be significant (*p* < 0.05) (Table 3).

Analysis of the MEDAS questionnaire scores showed significant increases in olive oil use, fruit portions, red/processed meat, legumes, and preference for poultry over red meat at course completion and 2 months after (*p* < 0.05). Vegetable portions, butter, margarine, cream, sugary drinks, and bakery product consumption also increased (*p* < 0.05). Sofrito use and nut consumption rose 2 months post-course (*p* < 0.05). No significant changes were observed in wine and seafood consumption (*p* > 0.05) (Table 3).

**Figure 2:**
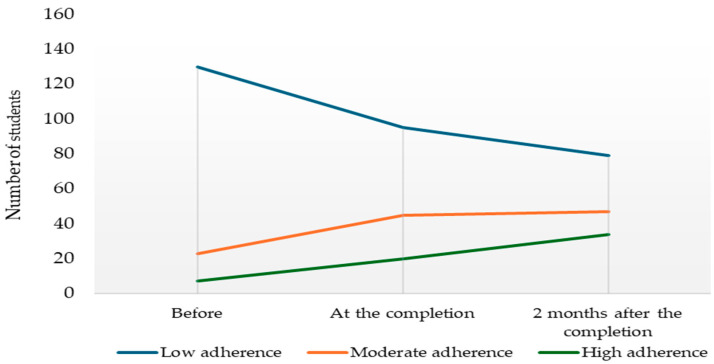
Changes in adherence to the MedDiet with education.

It is noteworthy that the number of those with low MedDiet adherence decreased with sustainability education. The number of students with moderate and high adherence increased with the education (Figure 2, Supplementary Materials Table S4).

When the MedDiet scores of the students were evaluated according to the department of study, it was found that the overall score of those studying in the Department of Nutrition and Dietetics was statistically significantly higher than that of those studying in other departments (Nursing, Child Development, Physiotherapy and Rehabilitation) (*p* < 0.05) (Supplementary Materials Table S5).

#### 3.3.2. HEI-2020 Scores of Students

The HEI-2020 total score increased significantly at the end of the course and 2 months after the course compared to before the course (*p* < 0.05). The increase between the time at which the course was completed and the time 2 months after the course was found to be significant (*p* < 0.05) (Table 4).

Statistically significant changes were observed in 6 of the 13 subcomponents of the HEI with sustainability education (Table 4).

Before the course, 73.8% of the students had poor diet quality. This dropped to 68.8% by the end of the course and further to 60.0% two months later (Supplementary Materials Table S6).

Students’ HEI-2020 scores were examined according to the students’ department, and it was found that there was no significant difference between the departments before the course, but at the completion of the course and 2 months after the course, the scores of the students in the Department of Nutrition and Dietetics were found to be statistically significantly higher compared to the others (*p* < 0.05) (Supplementary Materials Table S7).

### 3.4. Dietary Intake of Energy, Macronutrients, and Micronutrients by Students

Students’ total energy, fat (%), saturated fat, cholesterol, vitamin B12, and zinc intakes decreased statistically significantly (*p* < 0.05) during education. The educational process led to a statistically significant increase in the intake of carbohydrates (%), vegetable protein, dietary fiber, potassium, calcium, magnesium, and phosphorus (*p* < 0.05) (Table 5). Sustainability education did not affect intake of carbohydrates (g), protein (%), and iron (*p* > 0.05) (Table 5).

### 3.5. Environmental Impacts of University Students

Considering the students’ environmental impact, which changes with education, the total WFP, which is the sum of the green, blue, and grey WFP, decreased statistically significantly with education. CFP also decreased positively with the education process. (*p* < 0.05) (Table 6).

**Figure 3:**
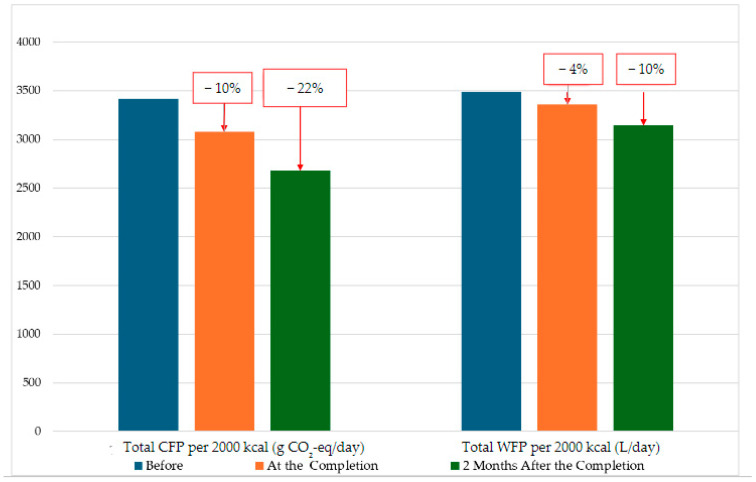
Changes in CFP and WFP with education.

CFP decreased by 10% at the end of the course and by 22% two months after the education compared to the beginning. Water footprint decreased by 4% at the end of the course and by 10% two months after the education compared to the beginning (Figure 3).

When the WFP and CFP values of the students were analyzed according to the department of study, it was observed that there was no significant difference between the departments throughout the courses (*p* > 0.05) (Supplementary Materials Tables S8 and S9).

## 4. Discussion

Bad eating habits are one of the important public health problems in the period of transition to university life. An increase in the consumption of fast food and cheaper food is one of the most common behaviors in university students. This situation emerges as important barriers to the adoption of healthy eating behaviors. Also, the lack of nutritional knowledge of university students draws attention [45].

This study basically aims to reveal the effect of sustainable nutrition education on diet quality, anthropometric measurements, and diet-related CFP and WFP. Studies evaluating the relationship between education, diet quality, and anthropometric measurements encourage healthy eating and physical activity. To the best of our knowledge, there is no study on the effect of sustainable nutrition courses on both diet quality and anthropometric measurements, especially in university students.

When the sustainability awareness levels of the students were evaluated, it was determined that 82 (51.2%) of them had not heard of this concept. Similarly, a survey of health professionals found that 47.1% had never heard of sustainable nutrition [46]. The majority of those who heard the concept of sustainability, 50.0% (*n* = 39) stated that they heard it from social media. In a further study on this topic, people who had heard of sustainable nutrition were reported to have first come across the concept through social media [47]. In both studies, social media stands out as the most common platform for hearing about sustainable nutrition. The power of social media in spreading the concept of sustainability is remarkable.

The literature shows that nutrition education positively affects anthropometric measurements [48,49]. At the same time, it has been observed that increased sustainable nutrition behaviors are associated with lower BMI levels [11]. In our study, it was found that there was a statistically significant decrease in weight, BMI, body fat ratio, and neck circumference in university students with sustainable nutrition education courses (*p* < 0.05). At this point, an education program that aims not only to increase the “general nutritional knowledge level” but also to address the environmental impact of food and to prevent food waste has similarly positive effects on anthropometric measurements and has potential effects on maintaining body weight and reducing the prevalence of obesity. 

A study evaluating the relationship between anthropometric measurements and nutrition education stated that 14-week nutrition education is a cost-effective and at the same time effective method to improve body composition and eating behavior in university students. As a result of the study, especially the increase in muscle mass was also noted [48]. In our study, no statistically significant difference was found in muscle mass during the courses (*p* > 0.05). Considering that the course duration was shorter and sustainable nutrition education aimed to reduce the consumption of animal-derived foods rich in protein, it is a possible result that there was no increase in muscle mass.

In our study, students in the Nursing and Physiotherapy and Rehabilitation departments had higher muscle mass and neck circumference compared to other departments. This is thought to be related to the fact that approximately 25% of the students studying in nursing and physiotherapy and rehabilitation departments are male.

Increased nutritional knowledge in university students has been shown to increase adherence to the Mediterranean diet and thus may be effective in reducing BMI and reducing the prevalence of obesity [50]. As a result of this study, it is thought that increased adherence to the MedDiet may be effective as the main reason for the decrease in BMI.

In this study, compared to the pre-education period, there was an increase of 1.86 points in the MedDiet score 2 months after the course was completed. It was found that multidisciplinary training, including nutrition education, could increase the MedDiet adherence score by 2.5 points and provide a decrease in body weight [49]. In another study, the MedDiet score increased by about 3 points after 8 weeks of nutritional training [51]. In a study conducted on adults, nutrition education was provided through an online platform to increase adherence to the MedDiet. As a result of the study, it was observed that the MedDiet score increased by 2.01 points in the 6th month and 2.67 points in the 12th month [52]. Considering that the follow-up period in this study was shorter and there was no multidisciplinary training, it is thought that the increase in the Mediterranean diet score is likely to be less.

Research conducted among university students showed that 76.4% of students had low adherence to the Mediterranean diet [53]. While 81.2% of the students had low adherence to the MedDiet at the beginning of this study, this rate decreased to 59.4% at the end of the course and to 49.4% 2 months after the course. In the previous study, it was observed that increased nutritional knowledge increases adherence to the MedDiet [54]. Considering the low adherence to the MedDiet among university students, not only general nutrition education but also sustained nutrition education has potential positive effects on increasing adherence.

One of the important ways to assess diet quality is the HEI. More sustainable dietary behaviors were found to have higher HEI-2015 scores compared to less sustainable dietary behaviors (62.6 vs. 51.9 points) [55]. In this study, the HEI score was 40.68 ± 13.36 before the course, and it increased by approximately 8 points two months after the education program was completed. In the study by Curi-Quinto et al., the sample consisted of individuals aged 18–59 years. Since the population of this study included university students, the HEI score is thought to be lower. In another study, it was observed that students educated in the department of nutrition and dietetics had HEI scores 13 points higher when they completed their course compared to students who did not complete their course [56]. When compared to 4 years of vocational education, the increase in the HEI score provided by short-term sustainable nutrition education is hopeful.

In our study, it was observed that the scores of MedDiet and HEI-2020, which are among the methods of evaluating diet quality, were higher in the students of the Department of Nutrition and Dietetics compared to the other departments when the course was completed and 2 months after the course. The main reason for this situation is thought to be related to the higher level of nutritional knowledge of nutrition and dietetics students due to the vocational education they have received. A study conducted in students living in the Marmara region, which is the region where this study was conducted, and studying in a nutrition and dietetics department found that the mean HEI-2020 score of the students was 49.3 ± 10.4 [24]. In our study, while the HEI-2020 score for the Department of Nutrition and Dietetics students was 43.81 ± 14.11 before the course, it increased about 15 points two months after the course. The effect of sustainability education on improving diet quality among those receiving nutrition education is remarkable.

In general, it is thought that increasing the consumption of plant foods and eliminating animal foods from the diet may lead to a decrease in protein, calcium, potassium, magnesium, zinc, and vitamin B12 intake; on the other hand, consumption of bakery products which contain saturated fat and sugar may increase [57]. In our study, there was no statistically significant difference in protein intake (g and %) with education. Consumption of plant protein sources increased by approximately 16.9%. It was observed that saturated fat intake, which is an important cause of concern, decreased, while calcium, potassium and magnesium intakes increased with sustainability education. As a result of this study, it is seen as an important fact that more sustainable eating behaviors cause a decrease in vitamin B12 and zinc intake. In conclusion, sustainability education has positive effects on dietary pattern except for vitamin B12 and zinc and does not affect total protein intake.

Sustainable eating behaviors aim to reduce the consumption of animal foods and increase the consumption of plant foods such as fruits, vegetables, whole grain products, and legumes [58]. When the MEDAS questionnaire is evaluated, reducing the consumption of animal foods such as red meat and processed meat or preferring poultry to red meat and consuming more fruits, vegetables, and legumes increases the total score [32]. Regarding the HEI-2020 score, an increase in fruit and vegetable consumption, plant protein sources, and whole grain intake results in an increase in the total score [33]. At this point, because of the increase in individuals’ sustainable nutrition behaviors with education, the increase in the MedDiet and HEI-2020 scores shows the effectiveness of education.

The food production process is responsible for the use of up to 80% of fresh water resources. Therefore, the WFP has become prominent in assessing environmental impact. The WFP is assessed by the Water Footprint Network in three different categories: green, blue, and grey WFP. Green WFP is the amount of rainwater required to produce a food or product. Blue WFP is the amount of surface or groundwater required to produce a food or product. Grey WFP is the amount of freshwater used to dilute pollutants resulting from the production of a food or product. All these categories make up the total WFP [59].

The vegetarian diet model, known as one of the sustainable diet models, had an average water footprint of 2655 L for 2000 kcal [60]. In our study, although the water footprint value decreased by 10% to 3141.42 ± 626.82 L two months after the course, it is thought that longer education is needed to reach the water footprint values of sustainable diet models.

A study conducted on university students in the USA on this topic found the carbon footprint of the diet to be 5209 g CO_2_-eq/day. As a result of sustainability education, the carbon footprint of these students decreased to 4077 g CO_2_-eq, a total decrease of 14% [61]. In our study, the pre-training carbon footprint was 3419.36 ± 1400.02 g CO_2_-eq, which decreased to 2677.88 ± 802.48 g CO_2_-eq 2 months after the training, a decrease of 22%. Considering that the population in our study was students from the Faculty of Health Sciences and that the students mostly live with their families at home, it is thought that the effect of education on food choices may be more prominent.

In our study, it would normally be expected that the CFP and WFP values of students who received nutrition education as a requirement of their profession would make a difference, but no difference was observed. A possible reason for this situation is that the CFP and WFP values were adjusted to 2000 kcal. Considering that the energy intake of nutrition and dietetics students is generally lower [62,63], it is thought that there may be differences in the data obtained without adjustment.

The greenhouse gas emission of Turkey’s national diet was reported as 3210 g CO_2_-eq/day and WFP as 2832 ± 1378 L/day [64]. It is seen that students have higher CFP and WFP values compared to the national diet before education. A study evaluating the diet-related WFP of university students in Turkey found it to be 3901 L/day [65]. It is seen that university students have more negative effects on the environment than the general population. It has been revealed that it is necessary to increase the level of knowledge of students, and it is thought that nutrition education can be an important solution to reduce the greenhouse gas emission and WFP values for the national diets of countries.

This study has some limitations. The main limitations of the research are that the sample of the research consists of only university students, the sample size is limited and mostly includes female students, the study is conducted in a certain period of time, and the results obtained are based on the answers of the participants. In the calculation of carbon footprint and water footprint, the deficiencies in the databases on food produced in Turkey are remarkable. For this reason, another limitation of the research is the use of global averages in some calculations.

## 5. Conclusions

The results of the study showed that sustainable nutrition education has the potential to improve diet quality while reducing weight, body mass index, BMI, and neck circumference independently of the department of study. Sustainable nutrition education was found to increase intakes of calcium, potassium, and magnesium while not altering total protein intake and decreasing intakes of B12 and zinc. Sustainability education among university students and other populations is potentially promising for both improving diet quality and combating obesity.

In addition, the effects of sustainability education on reducing diet-related CFP and WFP are encouraging in terms of preventing global warming, protecting clean water resources, and increasing planetary well-being.

## Figures and Tables

**Table 1:** Demographic characteristics and sustainability awareness levels of students.

	*n*	%
Gender		
Female	137	85.6
Male	23	14.4
	Mean ± SD	Min–Max
Age (Year)	21.13 ± 2.31	18–32
Department		
Nutrition and Dietetics	40	25.0
Child Development	40	25.0
Nursing	40	25.0
Physiotherapy and Rehabilitation	40	25.0
Class		
1st year	40	25.0
2nd year	40	25.0
3rd year	40	25.0
4th year	40	25.0
Living Status		
Family	134	83.8
Friends	8	5.0
Dormitory	18	11.2
Presence of Chronic Disease		
Yes	26	16.2
No	134	83.8
Medical Nutrition Therapy		
Yes	26	16.2
No	134	83.8
Type of Medical Nutrition Therapy		
Diabetes Diet	1	3.8
Low-Fiber Diet	1	3.8
Low-Fat, Low-Cholesterol Diet	1	3.8
Weight Loss Diet	23	88.5
Regular Medication Use		
Yes	0	0
No	160	100.0
Regular Supplement Use		
Yes	23	14.4
No	137	85.6
Has Heard About Sustainability		
Yes	78	48.8
No	82	51.2
Place Where Sustainability Was Heard About		
Books, newspapers, magazines, etc.	4	5.1
During undergraduate education	21	26.9
Scientific publications	2	2.6
Television and radio	3	3.8
Social media	39	50.0
Health professionals such as doctors, dieticians	9	11.5
Receiving Sustainability Courses		
Yes	15	9.4
No	145	90.6

SD, standard deviation; Min, minimum; Max, maximum.

**Table 2:** Anthropometric measurements of students during different phases of courses.

	Before ^1^	At the Completion ^2^	2 Months after the Completion ^3^	F	*p*-Value	Bonferroni	Eta Squared
	Mean ± SD	Mean ± SD	Mean ± SD				
Weight (kg)	63.80 ± 12.13	63.66 ± 12.01	63.57 ± 11.98	4.670	0.027 *	1 > 2, 3	0.029
BMI (kg/m^2^)	23.21 ± 3.61	23.15 ± 3.57	23.12 ± 3.54	5.892	0.012 *	1 > 2, 3	0.036
Body Fat (%)	25.26 ± 8.63	25.11 ± 8.42	25.0 ± 8.35	3.739	0.047 *	1 > 2, 3	0.023
Fat Mass (kg)	16.69 ± 7.83	16.53 ± 7.66	16.59 ± 7.89	0.546	0.478		
Muscle Mass (kg)	44.21 ± 7.39	44.31 ± 7.35	44.21 ± 7.13	0.252	0.622		
Neck Circumference (cm)	32.96 ± 3.76	32.94 ± 3.76	32.90 ± 3.75	5.252	0.017 *	1 > 2, 3; 2 > 3	0.032

BMI, body mass index; ^1^, before the course; ^2^, at the completion of the course; ^3^, two months after the completion of the course; SD, standard deviation; repeated measures ANOVA; Bonferroni test. * *p* < 0.05.

**Table 3:** MedDiet scores of students during different phases of courses.

	Before ^1^	At the Completion ^2^	2 Months after the Completion ^3^	F	*p*-Value	Bonferroni	Eta Squared
	Mean ± SD	Mean ± SD	Mean ± SD				
Olive oil as main fat	0.41 ± 0.49	0.51 ± 0.50	0.58 ± 0.50	11.592	0.000 *	1 < 2, 3; 2 < 3	0.068
Olive oil (≥4 ts/day)	0.18 ± 0.38	0.31 ± 0.47	0.36 ± 0.48	20.869	0.000 *	1 < 2, 3; 2 < 3	0.116
Vegetables (≥2 p/day)	0.24 ± 0.43	0.40 ± 0.49	0.43 ± 0.50	18.713	0.000 *	1 < 2, 3	0.105
Fruits (≥3 p/day)	0.06 ± 0.23	0.11 ± 0.31	0.16 ± 0.37	9.121	0.001 *	1 < 2, 3; 2 < 3	0.054
Red/processed meat (<1 p/day)	0.59 ± 0.49	0.69 ± 0.47	0.76 ± 0.43	12.337	0.000 *	1 < 2, 3; 2 < 3	0.072
Butter/margarine (<1 p/day)	0.66 ± 0.49	0.75 ± 0.43	0.78 ± 0.42	12.813	0.000 *	1 < 2, 3	0.075
Sugary beverages (<1 p/day)	0.59 ± 0.49	0.68 ± 0.47	0.70 ± 0.46	6.445	0.005 *	1 < 2, 3	0.039
Red wine (≥7 p/week)	0.00 ± 0.00	0.00 ± 0.00	0.00 ± 0.00	-	-		
Legumes (≥3 p/week)	0.19 ± 0.39	0.25 ± 0.43	0.31 ± 0.46	8.336	0.001 *	1 < 2, 3; 2 < 3	0.050
Fish/seafood (≥3 p/week)	0.02 ± 0.136	0.04 ± 0.19	0.05 ± 0.22	2.396	0.098		
Pastry products (<3 times/week)	0.36 ± 0.482	0.48 ± 0.501	0.53 ± 0.50	9.533	0.001 *	1 < 2, 3	0.057
Nuts (≥1 p/week)	0.56 ± 0.50	0.59 ± 0.49	0.67 ± 0.47	5.637	0.009 *	1 < 3; 2 < 3	0.034
Poultry products to red meat	0.27 ± 0.45	0.41 ± 0.49	0.51 ± 0.50	19.318	0.000 *	1 < 2, 3; 2 < 3	0.108
Sofrito (≥2 times/week)	0.56 ± 0.50	0.60 ± 0.49	0.66 ± 0.47	6.669	0.004 *	1 < 3; 2 < 3	0.040
Total MedDiet Score	4.63 ± 2.14	5.81 ± 2.27	6.49 ± 2.33	108.270	0.000 *	1 < 2, 3; 2 < 3	0.405

ts, table spoon; p, portion; MedDiet, Mediterranean diet; ^1^, before the course; ^2^, at the completion of the course; ^3^, two months after the completion of the course; SD, standard deviation; repeated measures ANOVA; Bonferroni test. * *p* < 0.05.

**Table 4:** HEI-2020 scores of students during different phases of courses.

	Before ^1^	At the Completion ^2^	2 Months after the Completion ^3^	F	*p*-Value	Bonferroni	Eta Squared
	Mean ± SD	Mean ± SD	Mean ± SD				
Total fruits	1.58 ± 1.86	2.02 ± 1.87	2.68 ± 1.76	20.690	0.000 *	1 < 2, 3; 2 < 3	0.116
Whole fruits	1.89 ± 2.27	2.59 ± 2.33	3.36 ± 2.10	26.617	0.000 *	1 < 2, 3; 2 < 3	0.143
Total vegetables	2.23 ± 1.53	2.30 ± 1.58	2.53 ± 1.54	2.631	0.075		
Greens and beans	1.58 ± 1.92	1.90 ± 2.13	2.44 ± 2.16	9.855	0.000 *	1 < 3; 2 < 3	0.058
Whole grains	4.19 ± 4.08	5.31 ± 4.03	5.55 ± 3.93	7.727	0.001 *	1 < 2.3	0.046
Dairy	3.23 ± 2.59	4.11 ± 2.88	4.95 ± 2.55	25.256	0.000 *	1 < 2, 3; 2 < 3	0.137
Total protein foods	2.91 ± 0.74	2.97 ± 0.66	2.86 ± 0.59	1.423	0.243		
Seafood and plant proteins	0.87 ± 1.71	1.05 ± 1.82	1.08 ± 1.75	1.030	0.355		
Fatty acids	3.28 ± 3.37	2.94 ± 3.45	3.66 ± 3.53	2.162	0.120		
Refined grains	3.74 ± 3.96	3.39 ± 4.13	3.89 ± 4.29	0.945	0.385		
Sodium	3.82 ± 3.90	3.26 ± 3.37	3.12 ± 3.30	2.218	0.114		
Added sugars	9.19 ± 1.51	9.26 ± 1.24	9.22 ± 1.20	0.159	0.843		
Saturated fats	2.17 ± 3.03	2.74 ± 3.17	3.41 ± 3.21	7.546	0.001 *	1 < 3; 2 < 3	0.045
Total HEI-2020 Score	40.68 ± 13.36	43.83 ± 13.70	48.06 ± 14.55	18.418	0.000 *	1 < 2, 3; 2 < 3	0.104

HEI-2020, Healthy Eating Index-2020; ^1^, before the course; ^2^, at the completion of the course; ^3^, two months after the completion of the course; SD, standard deviation; repeated measures ANOVA; Bonferroni test. * *p* < 0.05.

**Table 5:** Energy, macronutrient, and micronutrient intakes of students during different phases of courses.

	Before ^1^	At the Completion ^2^	2 Months after the Completion ^3^	F	*p*-Value	Bonferroni	Eta Squared
	Mean ± SD	Mean ± SD	Mean ± SD				
Energy (kcal)	1514.32 ± 460.53	1429.19 ± 404.60	1432.24 ± 378.76	14.063	0.000 *	1 > 2, 3	0.081
CHO (g)	164.86 ± 61.61	164.38 ± 55.50	170.41 ± 52.96	1.923	0.153		
CHO (%)	44.23 ± 8.48	46.88 ± 8.18	48.90 ± 6.98	17.670	0.000 *	1 < 2, 3; 2 < 3	0.100
Protein (g)	61.13 ± 22.81	59.68 ± 19.24	57.69 ± 16.46	2.545	0.083		
Plant Protein (g)	23.27 ± 9.84	25.48 ± 9.70	28.01 ± 9.84	19.101	0.000 *	1 < 2, 3; 2 < 3	0.107
Protein (%)	16.66 ± 4.23	17.31 ± 4.0	16.73 ± 3.23	1.840	0.162		
Fat (%)	38.94 ± 8.46	35.71 ± 7.58	34.56 ± 6.78	17.900	0.000 *	1 > 2, 3	0.101
Fiber (g)	15.09 ± 6.66	17.34 ± 7.00	19.96 ± 7.42	34.808	0.000 *	1 < 2, 3; 2 < 3	0.180
SFA (g)	27.78 ± 12.50	23.56 ± 9.89	22.68 ± 9.77	23.562	0.000 *	1 > 2, 3	0.129
Cholesterol (mg)	297.42 ± 169.05	267.53 ± 160.10	261.94 ± 150.01	3.761	0.025 *	1 > 2, 3	0.023
Vitamin B12 (mcg)	4.11 ± 4.85	3.49 ± 3.55	2.98 ± 1.43	4.417	0.019 *	1 > 3	0.027
Potassium (mg)	1852.72 ± 668.70	1877.19 ± 570.42	2086.84 ± 561.69	13.244	0.000 *	1 < 3; 2 < 3	0.077
Calcium (mg)	619.85 ± 302.01	647.58 ± 268.79	716.70 ± 243.02	10.464	0.000 *	1 < 3; 2 < 3	0.062
Magnesium (mg)	222.43 ± 81.19	231.76 ± 68.19	255.19 ± 71.85	16.100	0.000 *	1 < 3; 2 < 3	0.092
Phosphorus (mg)	931.28 ± 325.94	947.27 ± 277.06	1024.15 ± 302.21	8.758	0.000 *	1 < 3; 2 < 3	0.052
Iron (mg)	8.57 ± 3.63	8.20 ± 2.65	8.53 ± 2.63	1.192	0.299		
Zinc (mg)	9.19 ± 3.43	8.65 ± 2.85	8.33 ± 2.27	7.108	0.001 *	1 > 2, 3	0.043

kcal, kilocalorie; CHO, carbohydrate; SFA, saturated fatty acid; ^1^, before the course; ^2^, at the completion of the course; ^3^, two months after the completion of the course; SD, standard deviation; repeated measures ANOVA; Bonferroni test. * *p* < 0.05.

**Table 6:** CFP and WFP of students during different phases of courses.

	Before ^1^	At the Completion ^2^	2 Months after the Completion ^3^	F	*p*-Value	Bonferroni	Eta Squared
	Mean ± SD	Mean ± SD	Mean ± SD				
Green WFP per 2000 kcal (L/day)	2874.40 ± 941.22	2727.94 ± 771.52	2521.48 ± 585.06	9.406	0.000 *	1 > 3 2 > 3	0.056
Blue WFP per 2000 kcal (L/day)	349.52 ± 83.22	359.88 ± 88.16	348.96 ± 75.84	0.945	0.389		
Grey WFP per 2000 kcal (L/day)	263.22 ± 60,02	268.44 ± 61.22	262.92 ± 47.94	0.615	0.539		
Total WFP per 2000 kcal (L/day)	3487.14 ± 1015.58	3356.38 ± 852.52	3141.42 ± 626.82	7.831	0.001 *	1 > 3 2 > 3	0.047
Total CFP per 2000 kcal (g CO_2_-eq/day)	3419.36 ± 1400.02	3078.36 ± 1224.88	2677.88 ± 802.48	18.582	0.000 *	1 > 2, 3 2 > 3	0.105

L, liter; g, gram; eq, equivalent; kcal, kilocalorie; WFP, water footprint; CFP, carbon footprint; ^1^, before the course; ^2^, at the completion of the course; ^3^, two months after the completion of the course; SD, standard deviation; repeated measures ANOVA; Bonferroni test. * *p* < 0.05.

## Data Availability

The data that support the findings of this study will be made available by the corresponding author upon reasonable request. Data is not publicly available due to privacy and ethical concerns.

## Supplementary Materials

The following supporting information can be downloaded at: https://www.mdpi.com/article/10.3390/nu16111700/s1, Table S1: Anthropometric measurements of students according to department (before the course); Table S2: Anthropometric measurements of students according to department (at the completion of the course); Table S3: Anthropometric measurements of students according to department (2 months after the completion of the course); Table S4: Classification of students according to adherence to the MedDiet during different phases of courses; Table S5: Students’ MedDiet Scores according to department during different phases of courses; Table S6: Classification of students’ Healthy Eating Index-2020 scores during different phases of courses; Table S7: Healthy Eating Index-2020 scores of students according to department during different phases of courses; Table S8: CFP values of students according to department during different phases of courses; Table S9: WFP values of students according to department during different phases of courses; Excel worksheet for calculation of WFP and CFP.
