# Supplementary material for: The Impact of Sustainability Courses: Are They Effective in Improving Diet Quality and Anthropometric Indices?
Source: Nutrients. 2024 May 30;16(11):1700. doi: 10.3390/nu16111700 (PMC11174470; doi:10.3390/nu16111700)
Supplement: Supplementary file 1 [file nutrients-16-01700-s001.zip › Table S1-S9- ÇPF-SY-final version.pdf]

Table S1: Anthropometric Measurements of Students According to the Department (Before the Course)

	Department	n	Mean±SD	F	*p*-Value	Difference
Weight (kg)	Nutrition and Dietetics	40	60.205±7.971	2.157	0.095	
	Child Development	40	63.237±11.898			
	Nursing	40	66.712±13.409			
	Physiotherapy and Rehabilitation	40	65.060±13.838			
BMI (kg/m²)	Nutrition and Dietetics	40	22.622±2.951	0.576	0.631	
	Child Development	40	23.595±4.148			
	Nursing	40	23.475±3.277			
	Physiotherapy and Rehabilitation	40	23.147±3.985			
Body Fat (%)	Nutrition and Dietetics	40	26.323±7.291	1.711	0.167	
	Child Development	40	27.115±9.157			
	Nursing	40	23.255±8.117			
	Physiotherapy and Rehabilitation	40	24.348±9.530			
Fat Mass (kg)	Nutrition and Dietetics	40	16.378±6.613	0.657	0.580	
	Child Development	40	18.104±8.845			
	Nursing	40	15.726±6.673			
	Physiotherapy and Rehabilitation	40	16.565±8.965			
Muscle Mass (kg)	Nutrition and Dietetics	40	41.062±2.471	7.283	0.000 *	3>1<br>4>1<br>3>2
	Child Development	40	42.613±4.559			
	Nursing	40	47.792±10.281			
	Physiotherapy and Rehabilitation	40	45.380±7.917			
Neck Circumference(cm)	Nutrition and Dietetics	40	31.350±1.791	5.489	0.001 *	3>1<br>4>1<br>3>2<br>4>2
	Child Development	40	32.375±2.628			
	Nursing	40	34.075±4.671			
	Physiotherapy and Rehabilitation	40	34.050±4.495			

1, Nutrition and Dietetics; 2, Child Development; 3, Nursing; 4, Physiotherapy and Rehabilitation; BMI, Body Mass Index; SD, Standard deviation; One-Way ANOVA. * $p < 0.05$.

Table S2: Anthropometric Measurements of Students According to the Department (At the Completion of the Course)

	Department	n	Mean±SD	F	p-Value	Difference
Weight (kg)	Nutrition and Dietetics	40	59.825±7.756	2.314	0.078	
	Child Development	40	63.360±11.962			
	Nursing	40	66.460±13.317			
	Physiotherapy and Rehabilitation	40	64.998±13.504			
BMI (kg/m²)	Nutrition and Dietetics	40	22.465±2.880	0.788	0.502	
	Child Development	40	23.633±4.184			
	Nursing	40	23.378±3.211			
	Physiotherapy and Rehabilitation	40	23.120±3.870			
Body Fat (%)	Nutrition and Dietetics	40	25.962±7.067	1.814	0.147	
	Child Development	40	27.087±9.036			
	Nursing	40	23.045±7.778			
	Physiotherapy and Rehabilitation	40	24.337±9.335			
Fat Mass (kg)	Nutrition and Dietetics	40	16.033±6.416	0.870	0.458	
	Child Development	40	18.117±8.868			
	Nursing	40	15.495±6.394			
	Physiotherapy and Rehabilitation	40	16.481±8.640			

Muscle Mass (kg)	Nutrition and Dietetics	40	41.142±2.380	7.397	0.000 *	3>1<br>4>1<br>3>2
	Child Development	40	42.735±4.472			
	Nursing	40	47.910±10.254			
	Physiotherapy and Rehabilitation	40	45.445±7.883			
Neck Circumference (cm)	Nutrition and Dietetics	40	31.300±1.757	5.550	0.001 *	3>1<br>4>1<br>3>2<br>4>2
	Child Development	40	32.375±2.628			
	Nursing	40	34.025±4.677			
	Physiotherapy and Rehabilitation	40	34.050±4.495			

1, Nutrition and Dietetics; 2, Child Development; 3, Nursing; 4, Physiotherapy and Rehabilitation; BMI, Body Mass Index; SD, Standard deviation; One-Way ANOVA. * $p < 0.05$.

Table S3: Anthropometric Measurements of Students According to the Department (2 Months After the Completion of the Course)

	Department	n	Mean±SD	F	*p*-Value	Difference
Weight (kg)	Nutrition and Dietetics	40	59.618±7.579	2.423	0.068	
	Child Development	40	63.360±12.066			
	Nursing	40	66.365±13.278			
	Physiotherapy and Rehabilitation	40	64.952±13.402			
BMI (kg/m^2)	Nutrition and Dietetics	40	22.395±2.807	0.866	0.460	
	Child Development	40	23.616±4.204			
	Nursing	40	23.335±3.169			
	Physiotherapy and Rehabilitation	40	23.115±3.839			
Body Fat (%)	Nutrition and Dietetics	40	25.628±6.849	1.788	0.152	
	Child Development	40	26.988±8.996			
	Nursing	40	22.843±7.694			
	Physiotherapy and Rehabilitation	40	24.538±9.364			
Fat Mass (kg)	Nutrition and Dietetics	40	15.752±6.253	1.037	0.378	
	Child Development	40	18.059±8.925			
	Nursing	40	15.328±6.319			
	Physiotherapy and Rehabilitation	40	17.208±9.481			
Muscle Mass (kg)	Nutrition and Dietetics	40	41.358±2.375	5.766	0.001 *	3>1<br>4>1<br>3>2
	Child Development	40	42.868±4.383			
	Nursing	40	47.150±9.977			
	Physiotherapy and Rehabilitation	40	45.480±7.899			
Neck Circumference (cm)	Nutrition and Dietetics	40	31.175±1.708	5.724	0.001 *	3>1<br>4>1
	Child Development	40	32.400±2.649			
	Nursing	40	33.975±4.693			
	Physiotherapy and Rehabilitation	40	33.975±4.429			

1, Nutrition and Dietetics; 2, Child Development; 3, Nursing; 4, Physiotherapy and Rehabilitation; BMI, Body Mass Index; SD, Standard deviation; One-Way ANOVA. * $p < 0.05$.

Table S4: Classification of Students According to Adherence to the MedDiet During Different Phases of Courses

	n	%
MedDiet Adherence (Before)		
Low Adherence	130	81.2
Moderate Adherence	23	14.4
High Adherence	7	4.4
MedDiet Adherence (At the Completion)		
Low Adherence	95	59.4
Moderate Adherence	45	28.1
High Adherence	20	12.5
MedDiet Adherence (2 Months After the Completion)		
Low Adherence	79	49.4
Moderate Adherence	47	29.4
High Adherence	34	21.2

MedDiet, Mediterranean Diet.

Table S5: Students' MedDiet Scores According to the Department During Different Phases of Courses

	Department	n	Mean±SD	F	*p*-Value	Difference
MedDiet Score (Before)	Nutrition and Dietetics	40	5.550±2.275	3.572	0.015 *	1>2<br>1>3<br>1>4
	Child Development	40	4.150±1.718			
	Nursing	40	4.425±2.171			
	Physiotherapy and Rehabilitation	40	4.400±2.158			
MedDiet Score (At the Completion)	Nutrition and Dietetics	40	7.425±1.999	10.638	0.000 *	1>2<br>1>3<br>1>4
	Child Development	40	5.350±1.994			
	Nursing	40	5.300±2.334			
	Physiotherapy and Rehabilitation	40	5.175±2.011			
MedDiet Score (2 Months After the Completion)	Nutrition and Dietetics	40	7.775±2.106	6.263	0.000 *	1>2<br>1>3<br>1>4
	Child Development	40	5.975±2.130			
	Nursing	40	6.325±2.526			
	Physiotherapy and Rehabilitation	40	5.875±2.102			

1, Nutrition and Dietetics; 2, Child Development; 3, Nursing; 4, Physiotherapy and Rehabilitation; MedDiet, Mediterranean Diet; SD, Standard deviation; One-Way ANOVA. * $p < 0.05$.

Table S6: Classification of Students' Healthy Eating Index-2020 Scores During Different Phases of Courses

	n	%
Classification of HEI-2020 Scores (Before)		
Poor	118	73.8
Needs Improvement	41	25.6
Good	1	0.6
Classification of HEI-2020 Scores (At the Completion)		
Poor	110	68.8
Needs Improvement	49	30.6
Good	1	0.6
Classification of HEI-2020 Scores (2 Months After the Completion)		

Poor	96	60.0
Needs Improvement	64	40.0
Good	0	0.0

HEI-2020, Healthy Eating Index-2020.

Table S7: Healthy Eating Index-2020 Scores of Students According to the Department During Different Phases of Courses

	Department	n	Mean±SD	F	*p*-Value	Difference
HEI-2020 Score (Before)	Nutrition and Dietetics	40	43.81±14.11	1.135	0.337	
	Child Development	40	39.93±13.40			
	Nursing	40	40.47±14.29			
	Physiotherapy and Rehabilitation	40	38.50±11.36			
HEI-2020 Score (At the Completion)	Nutrition and Dietetics	40	53.87±15.15	12.636	0.000 *	1>2<br>1>3<br>1>4
	Child Development	40	42.68±10.37			
	Nursing	40	40.78±11.07			
	Physiotherapy and Rehabilitation	40	37.97±12.47			
HEI-2020 Score (2 Months After the Completion)	Nutrition and Dietetics	40	58.90±13.61	12.375	0.000 *	1>2<br>1>3<br>1>4
	Child Development	40	44.37±13.41			
	Nursing	40	46.09±11.46			
	Physiotherapy and Rehabilitation	40	42.89±14.15			

1, Nutrition and Dietetics; 2, Child Development; 3, Nursing; 4, Physiotherapy and Rehabilitation; HEI-2020, Healthy Eating Index-2020; SD, Standard deviation; One-Way ANOVA. * $p < 0.05$.

Table S8: CFP Values of Students According to the Department During Different Phases of Courses

	Department	n	Mean±SD	F	*p*-Value	Difference
Total CFP per 2000 kcal (g CO_2-eq/day) (Before)	Nutrition and Dietetics	40	3224.12±890.26	1.584	0.195	
	Child Development	40	3700.58±1064.66			
	Nursing	40	3563.62±937.48			
	Physiotherapy and Rehabilitation	40	3460.42±1129.50			
Total CFP per 2000 kcal (g CO_2-eq/day) (At the Completion)	Nutrition and Dietetics	40	2910.66±1031.66	0.837	0.475	
	Child Development	40	304534±1100.88			
	Nursing	40	3328.98±1361.22			
	Physiotherapy and Rehabilitation	40	3028.42±1374.96			
Total CFP per 2000 kcal (g CO_2-eq/day) (2 Months After the Completion)	Nutrition and Dietetics	40	2636.94±652.88	1.348	0.261	
	Child Development	40	2837.52±832.22			
	Nursing	40	2741.96±800.14			
	Physiotherapy and Rehabilitation	40	2495.06±902.72			

CFP,carbon footprint; kcal, kilocalories; g, gram; eq, equivalent; SD, Standard deviation; One-Way ANOVA.

Table S9: WFP Values of Students According to the Department During Different Phases of Courses

	Department	n	Mean±SD	F	*p* Value	Difference
Total WFP per 2000 kcal (L/day) (Before)	Nutrition and Dietetics	40	3224.12±890.26	1.584	0.195	
	Child Development	40	3700.58±1046,66			
	Nursing	40	3563.62±937.48			
	Physiotherapy and Rehabilitation	40	3460.42±1129.50			
Total WFP per 2000 kcal (L/day) (At the Completion)	Nutrition and Dietetics	40	3258.96±330.18	0.342	0.795	
	Child Development	40	3336.12±814.64			
	Nursing	40	3447.36±923.56			
	Physiotherapy and Rehabilitation	40	3382.50±995.52			
Total WFP per 2000 kcal (L/day) (2 Months After the Completion)	Nutrition and Dietetics	40	2993.40±596.34	1.443	0.232	
	Child Development	40	3242.94±584.06			
	Nursing	40	3098.06±548.74			
	Physiotherapy and Rehabilitation	40	3231.64±748.82			

WFP, water footprint; kcal, kilocalories; L, liter; SD, Standard deviation; One-Way ANOVA.
